# A multidisciplinary clinical treatment of locally advanced rectal cancer complicated with rectovesical fistula: a case report

**DOI:** 10.1186/1752-1947-6-369

**Published:** 2012-10-29

**Authors:** Tiancheng Zhan, Lin Wang, Ming Li, Zhongwu Li, Yong Cai, Lin Shen, Jin Gu

**Affiliations:** 1 Key Laboratory of Carcinogenesis and Translational Research (Ministry of Education), Department of Colorectal Surgery, Peking University School of Oncology, Beijing Cancer Hospital, 52 Fu Cheng Lu, Beijing, Haidian District 100142, PR China; 2 Key Laboratory of Carcinogenesis and Translational Research (Ministry of Education), Department of Pathology, Peking University School of Oncology, Beijing Cancer Hospital, 52 Fu Cheng Lu, Beijing, Haidian District, 100142, PR China; 3 Key Laboratory of Carcinogenesis and Translational Research (Ministry of Education), Department of Radiotherapy, Peking University School of Oncology, Beijing Cancer Hospital, 52 Fu Cheng Lu, Beijing, 100142, Haidian District, PR China; 4 Key Laboratory of Carcinogenesis and Translational Research (Ministry of Education), Department of Gastrointestinal Oncology, Peking University School of Oncology, Beijing Cancer Hospital, 52 Fu Cheng Lu, Beijing, Haidian District, 100142, PR China

## Abstract

**Introduction:**

Rectal cancer with rectovesical fistula is a rare and difficult to treat entity. Here, we describe a case of rectal cancer with rectovesical fistula successfully managed by multimodality treatment. To the best of our knowledge, this is the first such case report in the literature.

**Case presentation:**

A 51-year-old Chinese man was diagnosed as having rectal cancer accompanied by rectovesical fistula. He underwent treatment with neoadjuvant radiochemotherapy combined with total pelvic excision and adjuvant chemotherapy, as recommended by a multimodality treatment team. Post-operative pathology confirmed the achievement of pathological complete response.

**Conclusions:**

This case suggests that a proactive multidisciplinary treatment is needed to achieve complete cure of locally advanced rectal cancer even in the presence of rectovesical fistula.

## Introduction

Most colorectal cancers in the upper rectum or sigmoid colon invade the top of the bladder, and they are clinically treated with a relatively simple en-bloc resection of the invaded bladder [1]. When the tumor is located in the anterior wall in the middle of the rectum it is likely to invade the bladder trigone [1], and when tumor is in the low rectum it is likely to invade the prostate and seminal vesicles. Thus, special handling is required for mid to low rectal cancer, especially in men.

## Case presentation

A 51-year-old Asian man presented to our facility with issues of increased stool frequency (six to eight times a day) accompanied with tenesmus for more than a month. The stool was shapeless and occasionally mixed with small amounts of blood. He was admitted due to worsening of these symptoms and fecaluria accompanied with fever and severe body weight loss.

**Figure 1:**
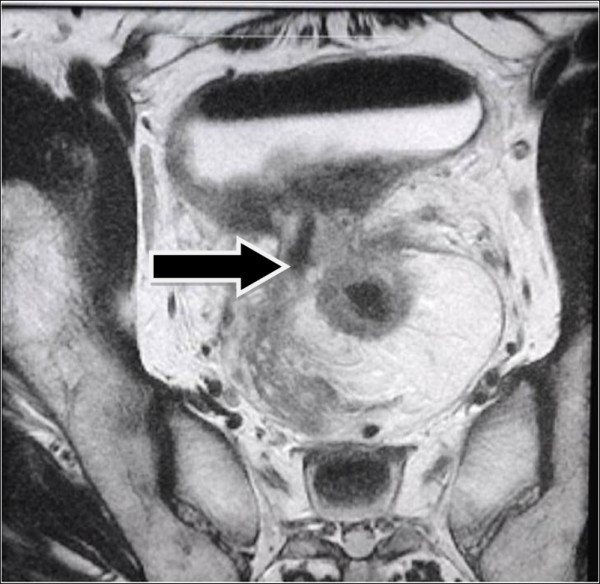
**Magnetic resonance image of rectal cancer prior to neoadjuvant therapy.** Arrow indicates the rectovesical fistula sinus.

**Figure 2:**
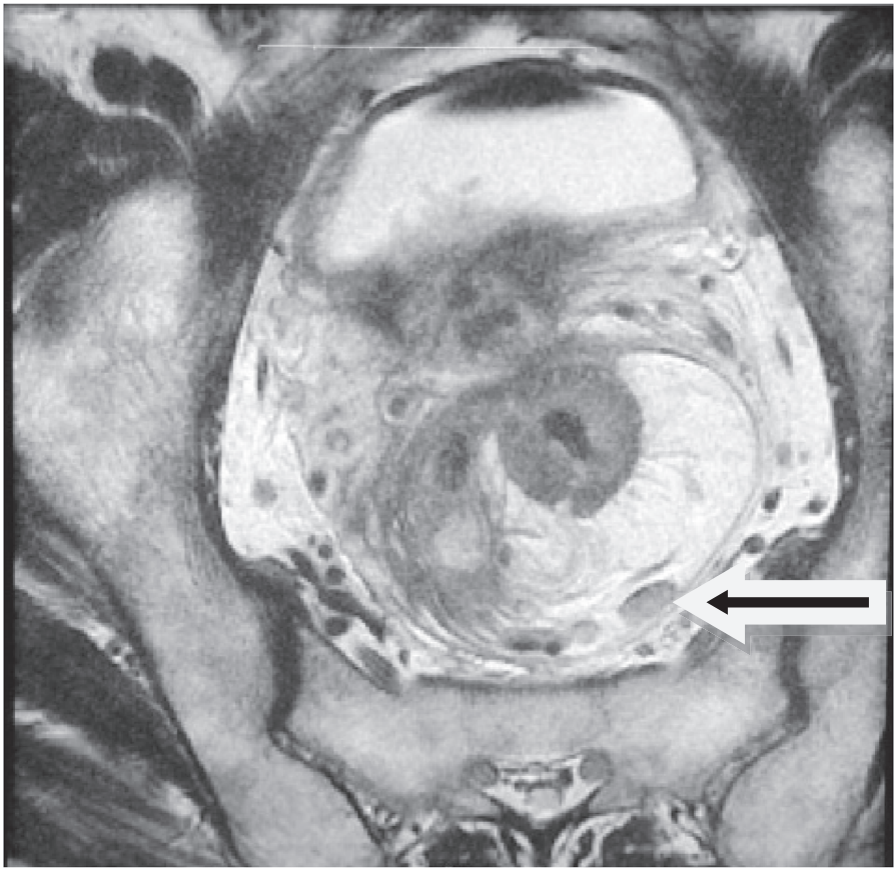
**Magnetic resonance image of rectal cancer prior to neoadjuvant therapy.** Arrow indicates the enlarged lymph nodes in mesorectum.

A protruding peri-rectal lump in the anterior wall with an uneven surface was found by digital rectal examination. A colonoscopy examination revealed a mucosal bulge 4cm from the anal verge with surface erosion and stenosis. A rectal biopsy confirmed the diagnosis of moderately differentiated adenocarcinoma. Pelvic computed tomography (CT) showed a rectal cancer that had invaded the bladder. Cystoscopy revealed a 6×6cm lump at the six o’clock position of the bladder neck and trigone with ulcerative erosion on the surface, suggesting an invasion of rectal tumor. Pelvic magnetic resonance imaging (MRI) showed extensive thickening of the wall in the upper rectum with a broken outer membrane by the lesion on the right side that spread to the bladder, where intestinal contents were observed (Figure 1). Multiple enlarged lymph nodes were observed next to iliac vessels inside the mesorectum (Figure 2). The results of a urine white blood cell test were positive. A chest X-ray and abdominal ultrasound did not show any distant metastases. Our patient was clinically diagnosed as having locally advanced rectal cancer, T4bN2M0, stage IIIC, complicated with rectovesical fistula, incomplete rectal obstruction, lower gastrointestinal bleeding, and pelvic local infection.

Given that our patient had incomplete intestinal obstruction, rectal bladder fistula, local hemorrhage and infection, radical resection of the tumor was not suitable. A multimodality treatment team consisting of surgeons, physicians, imaging scientists, pathologists and radiologists suggested conducting a transverse colostomy to bypass feces first, subsequently performing pre-operative neoadjuvant therapy when our patient became stable. A week after transverse colostomy, all his clinical symptoms disappeared and neoadjuvant chemotherapy was administrated using oxaliplatin 80mg once a week (50mg/m^2^) and capecitabine 1.5g twice a day (1000mg/m^2^) for four weeks, combined with 10MV X-ray intensity modulated radiation therapy (IMRT) of gross tumor volume (GTV) 50.6Gy/clinical target volume (CTV) 41.8Gy for 22 days.

**Figure 3:**
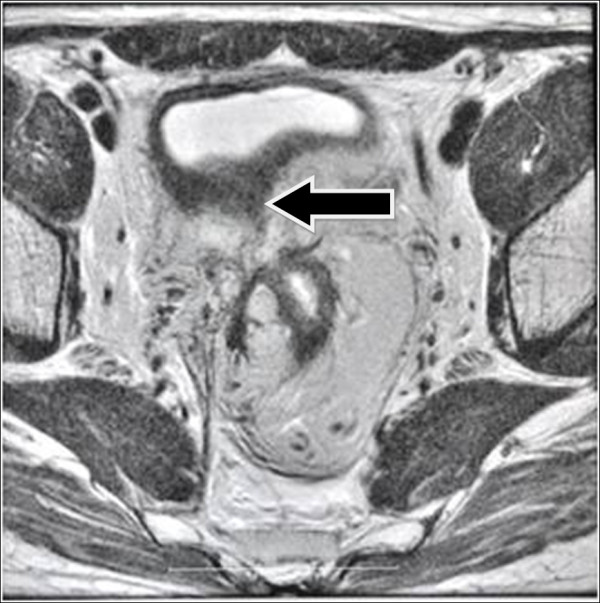
**Magnetic resonance image of rectal cancer after neoadjuvant radiotherapy.** The arrow indicates closure of rectovesical fistula.

**Figure 4:**
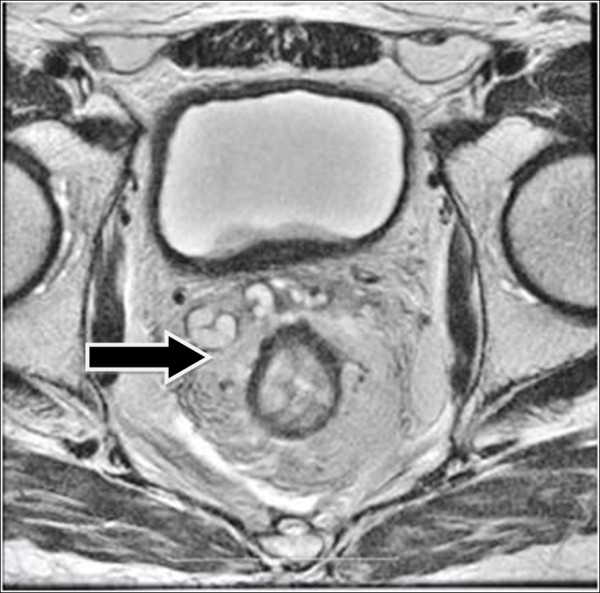
**Magnetic resonance image of rectal cancer after neoadjuvant radiotherapy.** The arrow indicates the reduced tumors. Some enlarged lymph nodes have disappeared.

Two months after neoadjuvant therapy, MRI reassessment showed that the thickness of the upper rectum wall was reduced with significant tumor reduction. The lower end of the tumor was located 4cm above the junction of the levator ani muscle and rectum with local invasion to the muscular layer of the wall. Inside the mesorectum, the size of the multiple enlarged lymph nodes next to iliac vessels had also reduced (Figure 3). The lump size localized on the right mesorectal fascia was significantly reduced. However, the irregular thickening of the posterior wall of the bladder was still observed (Figure 4). Positron emission tomography-computed tomography (PET-CT) confirmed a rectum-sigmoid junction cancer spreading to the bladder trigone with metastasis to local lymph nodes of the mesorectum, retroperitoneal and iliac vascular region. No distant metastasis was detected.

**Figure 5:**
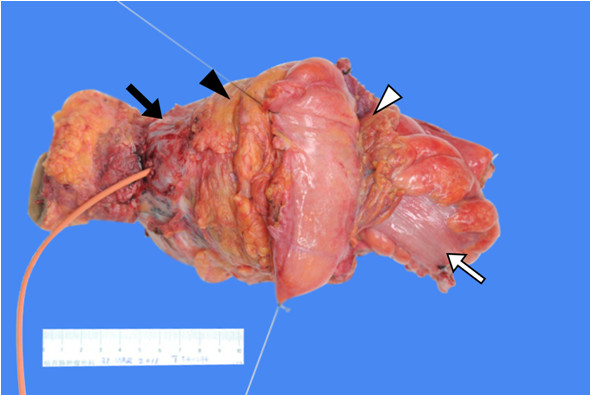
**Specimen of total pelvic exenteration surgery sutured at the peritoneal reflection.** ▹ Indicates the peritoneal reflection, ▸ indicates the mesorectal region, ➩ indicates the rectal region, ➡ indicates the invasion of rectal cancer through the bladder wall.

**Figure 6:**
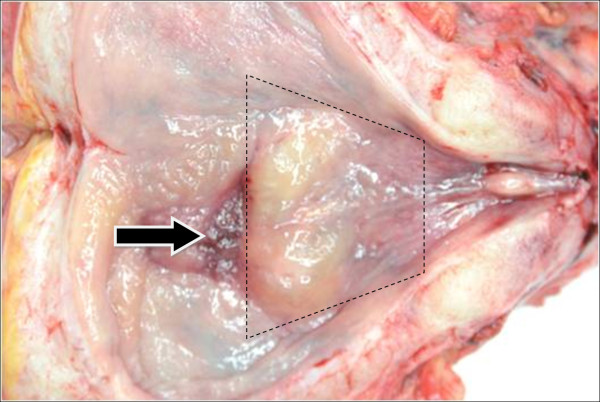
**Specimen of total pelvic exenteration surgery showing the triangle area of bladder.** The arrow indicates the invasion of rectal cancer through the bladder wall.

**Figure 7:**
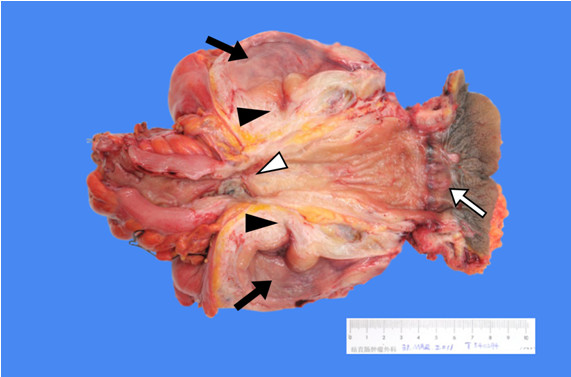
**Profile view of total pelvic exenteration surgery specimen.** ▹ Indicates the necrotic rectal cancer following chemoradiotherapy, ▸ indicates the closed rectovesical fistula, ➩ indicates the anal region, ➡ indicates the bladder mucosa.

**Figure 8:**
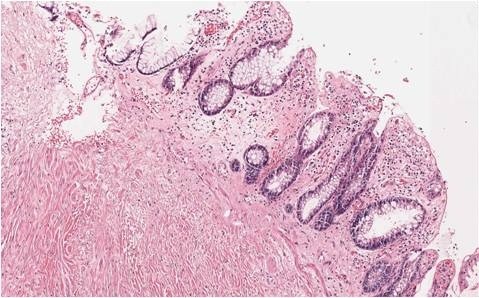
**Pathology of resected specimen.** Hematoxylin and eosin stain, ×100.

**Figure 9:**
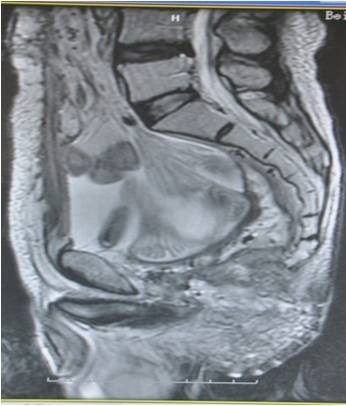
Sagittal section of magnetic resonance image after operation.

With clinical improvement of the rectovesical fistula after neoadjuvant therapy, a total pelvic exenteration (TPE) plus cutaneous ureterostomy was performed for better treatment of the invaded bladder and involved pelvic lymph nodes. Pathological analysis of the surgical specimens (Figures 5, 6, 7) revealed no residual cancer cells, lymphovascular invasion (LVI) or local lymph nodes metastasis (out of 22 lymph nodes), suggesting a pathological complete response (PCR) after neoadjuvant therapy (Figure 8). Five cycles of XELOX regimen (capecitabine plus oxaliplatin) were given as adjuvant therapy. One month after TPE, no abnormal enhancement sites and enlargement of lymph nodes were found in the pelvis by MRI (Figure 9).

## Discussion

According to the National Comprehensive Cancer Network (NCCN) guidelines, neoadjuvant therapy should be given for the treatment of locally advanced rectal cancer (LARC) [2]. However, for tumors that invade into the bladder trigone resulting in rectovesical fistula, the treatment becomes complicated. Therefore, a multidisciplinary clinical team is needed to find comprehensive solutions [3]. All the symptoms such as fever, blood in the stool, fecaluria, and incomplete intestinal obstruction present in our patient resulted from the bladder fistula caused by local invasion of rectal cancer. After under going the operation to create a diverting stoma, all his symptoms disappeared within a week, which helped our patient undergo further treatment.

The incidence of fecaluria caused by rectovesical fistula is relatively low in rectal cancer invading the bladder. Since simple rectal resection would not remove residual tumor in the bladder, we administered neoadjuvant chemotherapy using a XELOX regimen combined with 50.6Gy of pre-operative radiation [4,5]. The standard pre-operative treatment for locally invasive rectal cancer in our hospital included fractional radiation with cumulative 50.4Gy combined with oral administration of capecitabine. In addition to the standard pre-operative treatment, four weeks of oxaliplatin was also given in this case to enhance tumor killing and increase the sensitivity of radiation therapy. This treatment may also contribute to the control of distant metastasis caused by long-term pre-operative treatment. Reassessment after neoadjuvant therapy showed tumor shrinkage and absence of distant metastases. A reasonable therapeutic option could be the performance of a limited surgical procedure if cystoscopy excludes residual tumor in the bladder. Then, a strict urologic follow-up could show eventual vesical relapse and a secondary total cystectomy could be performed. However, we believe that pathological analysis is more accurate than cystoscopy for the diagnosis of residual tumor in the bladder. Considering the multiple enlarged lymph nodes and the good general condition of our patient to tolerate the operation, we performed TPE. Post-operative adjuvant therapy was carried out based on the opinion that post-operative adjuvant therapy is necessary even for a PCR of rectal cancer [2,4,5].

## Conclusions

The findings from our patient’s case suggest that a proactive multidisciplinary treatment is needed to achieve complete cure for locally advanced rectal cancer complicated with rectovesical fistula.

## Consent

Written informed consent was obtained from the patient for publication of this manuscript and any accompanying images. A copy of the written consent is available for review by the Editor-in-Chief of this journal.

## Competing interests

The authors declare that they have no competing interests.

## Authors’ contributions

TZ analyzed and interpreted the data from our patient regarding rectal cancer and the rectovesical fistula, and was a major contributor in writing the manuscript. LW took the picture for the specimen. ZL performed histological examination of the specimen. JG, ML and TZ performed the TPE for our patient. LS designed the chemotherapy regimen and YC designed the radiotherapy regimen for our patient. All authors read and approved the final manuscript.
